# Dictionary learning-based reverberation removal enables depth-resolved photoacoustic microscopy of cortical microvasculature in the mouse brain

**DOI:** 10.1038/s41598-017-18860-3

**Published:** 2018-01-17

**Authors:** Sushanth Govinahallisathyanarayana, Bo Ning, Rui Cao, Song Hu, John A. Hossack

**Affiliations:** 1 Department of Biomedical Engineering, University of Virginia, Charlottesville, VA 22908 USA; 2 Present Address: Department of Biomedical Engineering, Johns Hopkins University, Baltimore, MD 21205 USA

## Abstract

Photoacoustic microscopy (PAM) capitalizes on the optical absorption of blood hemoglobin to enable label-free high-contrast imaging of the cerebral microvasculature *in vivo*. Although time-resolved ultrasonic detection equips PAM with depth-sectioning capability, most of the data at depths are often obscured by acoustic reverberant artifacts from superficial cortical layers and thus unusable. In this paper, we present a first-of-a-kind dictionary learning algorithm to remove the reverberant signal while preserving underlying microvascular anatomy. This algorithm was validated *in vitro*, using dyed beads embedded in an optically transparent polydimethylsiloxane phantom. Subsequently, we demonstrated in the live mouse brain that the algorithm can suppress reverberant artifacts by 21.0 ± 5.4 dB, enabling depth-resolved PAM up to 500 µm from the brain surface.

## Introduction

Photoacoustic microscopy (PAM) capitalizes on the optical absorption of endogenous biomolecules (e.g. blood hemoglobin) to enable label-free high-resolution functional and molecular imaging *in vivo*^1–3^. By combining focused light excitation and time-resolved acoustic detection, PAM possesses three-dimensional resolving capability. However, the acoustic reverberation from superficial PAM signals often manifests as spurious ‘ghosting’ artifacts at depths^4^, thereby obscuring the underlying signal and severely compromising the depth-resolving capability of PAM. Utilizing a known point spread function or transform basis in which the data can be sparsely represented, sparsity-based methods have been applied to deconvolution, compressed sensing, denoising, and super-resolution in photoacoustic imaging^5–8^.

These methods utilize underlying spatial or temporal redundancy of the photoacoustic signal to improve the speed of acquisition or image quality. For example, compressed sensing^5,9^ is used to achieve higher acquisition speed and reconstruct high quality images from undersampled measurements of data which are sparse in a transform domain. Much of the data which is not sampled during the acquisition is noise as it is seldom sparse in the transform domain. Consequently, the final reconstructed images possess a higher signal to noise ratio (SNR) than achievable using conventional sampling. Hojman *et al*.^5^ formulated a compressed sensing method to reduce the number of images to be acquired in a photoacoustic tomography (PAT) system thus accelerated the imaging speed and reduced noise. The method also leads to super-resolved images, below the diffraction limit, by sparse deconvolution with the system response. Burgholzer *et al*.^6^ developed a sparsifying transformation using the connection between the spatial and temporal evolution of the photoacoustic wave and subsequently applied it to compressed sensing reconstruction in PAT.

These methods are mainly focused on accelerating image acquisition using a known point spread function (PSF) or basis in PAT systems while still achieving high quality reconstructions using non-linear iterative reconstruction methods such as L1 minimization, or total variation minimization. Our method focuses on reducing reverberant artifacts in PAM, without the need for a known PSF. We utilize a sparse model of the true signal and reverberant artifacts in PAM, and consequently suppress it by adaptively learning a basis in which the signal is sparse using Dictionary learning (DL) methods.

Dictionary learning (DL) adaptively learns a basis in which the signal is sparse and can be reconstructed faithfully using data with spatial or temporal redundancy. The data is encoded as a sparsely weighted combination of basis vectors that are adaptively learned from the data. Haq *et al*.^8^ utilize the K-SVD^10^ DL method to reduce noise present in Photoacoustic Microscopy (PAM) images using the approach outlined by Mairal *et al*.^11^. The approach utilizes local signal characteristics, and suppresses spatially incoherent stochastic noise using the averaging of overlapping patches. Spatially coherent reverberant artifacts are unlikely to be affected by this method, and consequently must be removed by a different method.

In our work, a signal model of reverberation in PAM as the convolution of the impulse response of ultrasonic transducer with a sparse spatial impulse response is developed. Consequently, DL is exploited to remove the reverberant artifacts. This differs from other sparsity-based methods described above in that the basis that describes the PAM signal is adaptively learned from the data, and reverberant artifacts are removed in addition to random noise. This paper is organized as follows: first, a sparse spatial impulse response model of reverberation is presented as a justification for the use of DL. Subsequently, the DL procedure is described and used to demonstrate the accurate coding of PAM signals. The basis vectors of the dictionary are reweighted to suppress reverberant artifacts while retaining true signals at depths. Finally, *in vitro* validation using a transparent polydimethylsiloxane (PDMS) phantom embedded with dyed polystyrene beads and *in vivo* validation in the live mouse brain are presented.

## Results

### Sparse reverberation model

The photoacoustic response of an absorber is the result of transient laser pulse-induced heating followed by thermoelastic expansion^2,12^. The response to a nanosecond pulse excitation, which is typically used in PAM, has been modeled as a spatial impulse by Diebold *et al*.^2^. The detected photoacoustic signal (*PA*_*o*_) from the original laser pulse is the convolution of the transducer receive mode impulse response *H* with this impulse.

1 $$P{A}_{o}=H\,\bigotimes\,\delta (t-\frac{z}{{v}_{s}}),$$

where ⨷ denotes the convolution operation, *H* the transducer receive mode impulse response, ***z*** the depth of the source, and ***v***_***s***_ the velocity of sound in the medium. Similarly, signals from a photoacoustic point source at a depth ***z*** contaminated with reverberant echoes may be modelled as follows.

**Figure 1:**
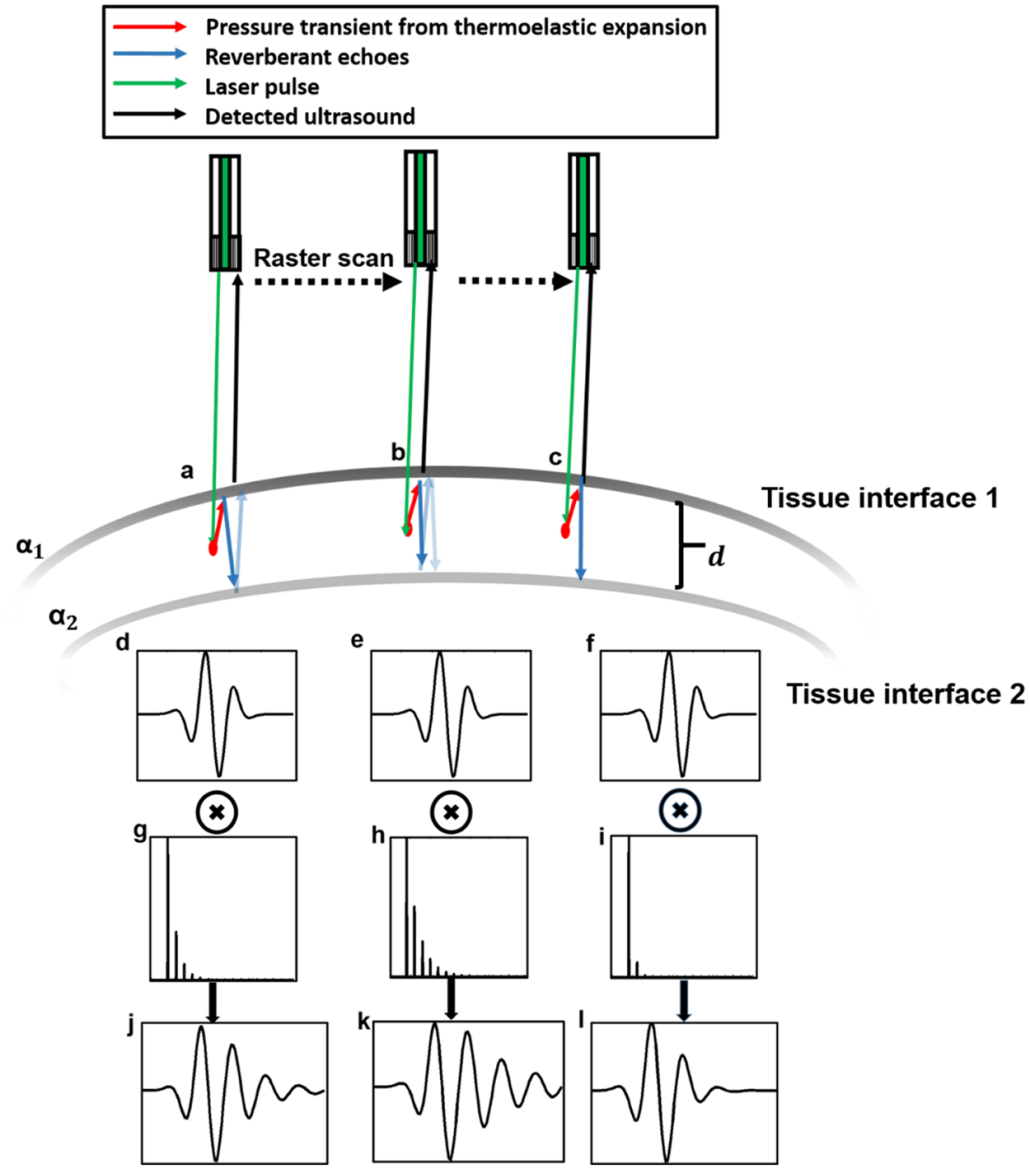
Schematic showing photoacoustic signals detected with different reverberation responses at 3 points (**a**–**c**) during a raster scan. (**d**–**f**) Transducer impulse response. (**g**–**i**) Reverberation responses. (**j**–**l**) Detected photoacoustic signals. ⨷ Denotes the convolution operator. The reverberant echoes are denoted with lighter arrows to indicate reducing intensity. *α*_1_, *α*_2_ denote the respective echogenicity of the two tissue interfaces separated by *d*.

Acoustic reverberation is characterized by periodically spaced echoes generated by repeated reflections between near-parallel interfaces in the medium. Consider a point photoacoustic source at depth *z* in tissue between the two interfaces (Fig. 1). Let the normalized echogenicity at the first tissue interface be ***α***_1_ and acoustic reflectance at the interface 2 at a depth ***d*** from the first interface be ***α***_2_. Hence, the fractions of energy transmitted through the interface 1 and 2 are 1−*α*_1_ and 1−*α*_2_, respectively. The received photoacoustic signal is a combination of the signal generated at the original point source and that from successively higher-order reverberant echoes. The signal at each order of reverberation is a superposition of reverberation from each of the parallel interfaces surrounding the point where the signal is produced. The photoacoustic signal contaminated with reverberant signal (*R*) can be expressed through the following series:

From interface 1,

$$\begin{array}{rcl}{R}_{T} & = & (1-{\alpha }_{1})H(t-\frac{z}{{v}_{s}})+{\alpha }_{1}{\alpha }_{2}(1-{\alpha }_{1})H(t-\frac{z+2d}{{v}_{s}})\\  &  & +{({\alpha }_{1}{\alpha }_{2})}^{2}(1-{\alpha }_{1})H(t-\frac{z+4d}{{v}_{s}}).\end{array}$$

or

2 $$\,{R}_{T}=\sum _{i=0}^{\infty }{({\alpha }_{1}{\alpha }_{2})}^{i}(1-{\alpha }_{1})H(t-\frac{z+2id}{{v}_{s}}).$$

And from interface 2,

$$\begin{array}{rcl}{R}_{B} & = & {\alpha }_{2}(1-{\alpha }_{1})H(t-\frac{z+d}{{v}_{s}})+{{\alpha }_{2}}^{2}{\alpha }_{1}(1-{\alpha }_{1})H(t-\frac{z+3d}{{v}_{s}})\\  &  & +{{\alpha }_{2}}^{3}{\alpha }_{1}^{2}(1-{\alpha }_{1})H(t-\frac{z+5d}{{v}_{s}}).\end{array}$$

or

3 $$\,{R}_{B}=\sum _{i=0}^{\infty }{{\alpha }_{2}}^{(i+1)}{\alpha }_{1}^{i}(1-{\alpha }_{1})H(t-\frac{z+(2i+1)d}{{v}_{s}}).$$

Combining Eqns (2) and (3), we have

4 $$\begin{array}{lll}R={R}_{B}+{R}_{T} & = & (1-{\alpha }_{1})\sum _{i=0}^{\infty }{({\alpha }_{1}{\alpha }_{2})}^{i}H(t-\frac{z+2id}{{v}_{s}})\\  &  & +({{\alpha }_{2}}^{i+1}{\alpha }_{1}^{i})H(t-\frac{z+(2i+1)d}{{v}_{s}}).\end{array}$$

It may be observed that since $\,{\alpha }_{1},{\alpha }_{2}\le 1$, higher-order terms are smaller in magnitude. Hence, in practical cases, the reverberant signal is either beyond the dynamic range of the PAM system or buried under the noise floor of the system after multiple reflections. To reflect this, the series may be truncated to a finite number of terms ($2N$). Using Eqns (1) Eqn (4) can be modified as

5 $$R=(1-{\alpha }_{1})H\,\bigotimes\,\sum _{i=0}^{N-1}{({\alpha }_{1}{\alpha }_{2})}^{i}\delta (t-\frac{z+2id}{{v}_{s}})+({{\alpha }_{2}}^{i+1}{\alpha }_{1}^{i})\delta (t-\frac{z+(2i+1)d}{{v}_{s}})\,.$$

Furthermore, since the arrival times of photoacoustic pulses are discrete, the reverberant echo is inherently sparse. Thus, *R* can be written as

6 $$R=PS\,{\rm{subject}}\,{\rm{to}}{\Vert S\Vert }_{0}\le 2N,$$

where $$[\begin{array}{c}(1-{\alpha }_{1})\\ \vdots \\ ({{\alpha }_{2}}^{N}{\alpha }_{1}^{N-1})(1-{\alpha }_{1})\end{array}]$$ is a sparse vector owing to the discrete arrival times of reverberant echoes and the number of reflections *N*, $||\,|{|}_{0}$ denotes the L0 pseudo norm^13–15^ (number of non-zero coefficients) In general, *S* represents the spatial impulse response of the medium dependent on the acoustic properties of the parallel interfaces causing the reverberation and *P* represents a matrix, where each column is the transducer receive mode impulse response shifted axially to the spatial location of the transient pressure

7 $$P=\bigcup _{i=0}^{2N-1}H(t-\frac{z+id}{{v}_{s}}).$$

**Figure 2:**
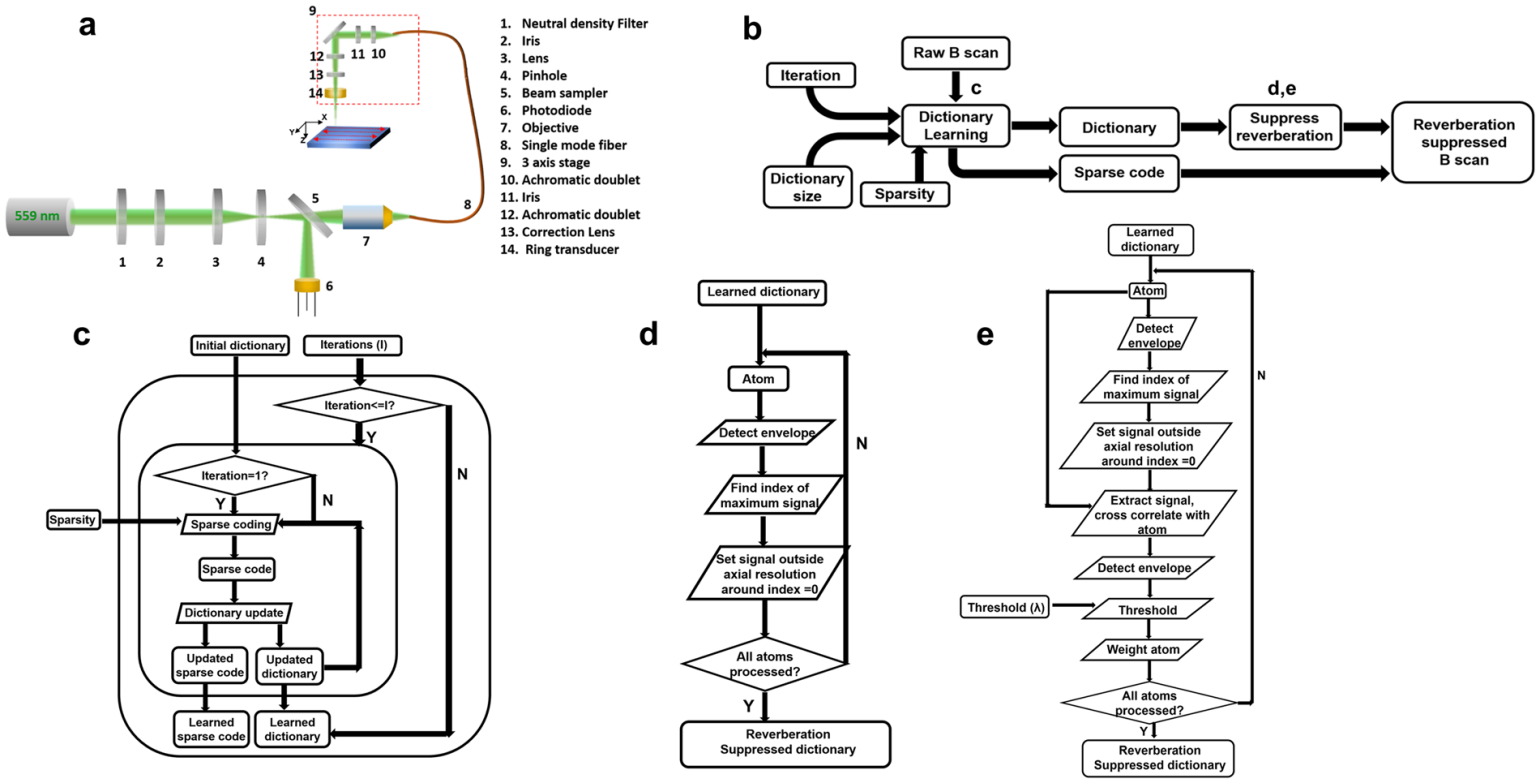
(**a**) Schematic of the PAM setup. (**b**) Reverberation suppression procedure. The white arrows indicate reverberant artifacts and the red arrows indicate true signals of small amplitude.

Note that it is not feasible to measure *S* experimentally, given the heterogeneity of tissue and structures within the body. It follows from Eqn. (6) that if the signal at a given depth in the tissue contains only the true signal but no reverberation, then $\,||S|{|}_{0}=1$. In this case, the signal at this point is only represented by a single copy of the transducer impulse response shifted to that point. PAM data are acquired as a two dimensional ensemble of A-lines by raster scanning the region of interest with coaxially and confocally aligned dual foci^16^ (Fig. 2a). An A-line is a combination of signals and reverberant echoes from multiple sources over the imaging depth. This can be represented as a sparse linear combination of basis vectors (Supplementary section S1). The number of possible basis vectors exceeds the dimensionality of the A-line, which makes this basis over-complete and consequently a vector of weights that combines these basis vectors is sparse. DL algorithms can adaptively learn over-complete bases and sparse weighting vectors simultaneously from the data. Once the basis is computed, it follows from Eqn. (6) that if we reduce the contribution of the reverberant responses in *S* to just the signal, i.e. with ${\Vert S\Vert }_{0}=1$. Then the reverberation is suppressed. The complete reverberation suppression algorithm (Fig. 2b) is illustrated in pseudo-code as follows.
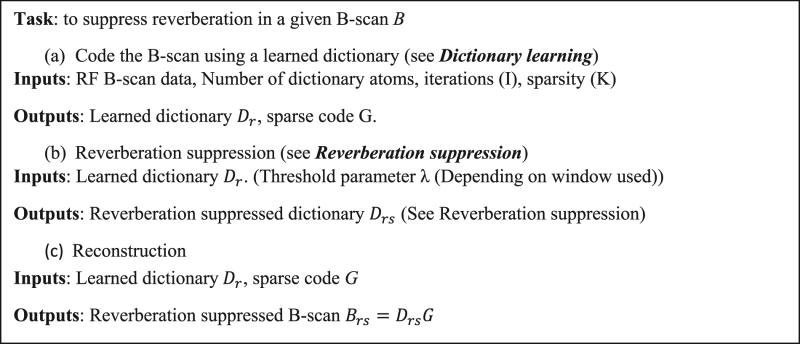

### Dictionary Learning

In the previous section, we have described a representation (Eqn. (6)) to characterize PAM data at a given depth in tissue. Volumes of PAM data are acquired as a two-dimensional ensemble of A-lines. Given the PAM data from a B-scan (*B*), it may be observed from our formulation Eqns (2–7) that *B* can be completely characterized if the matrices, *P* and *S* are known at all depths, which effectively computes the over-complete basis and sparse weighting described in Supplementary section S1. In the presence of system noise, an approximation to the basis and sparse weighting vector can be adaptively learned from the raw data using dictionary learning, which is formulated as the following optimization problem:

8 $$B=DG\,subject\,to\,{\parallel B-DG\parallel }_{2}\le \varepsilon \,,{\parallel G\parallel }_{0}\le K.$$

**Figure 3:**
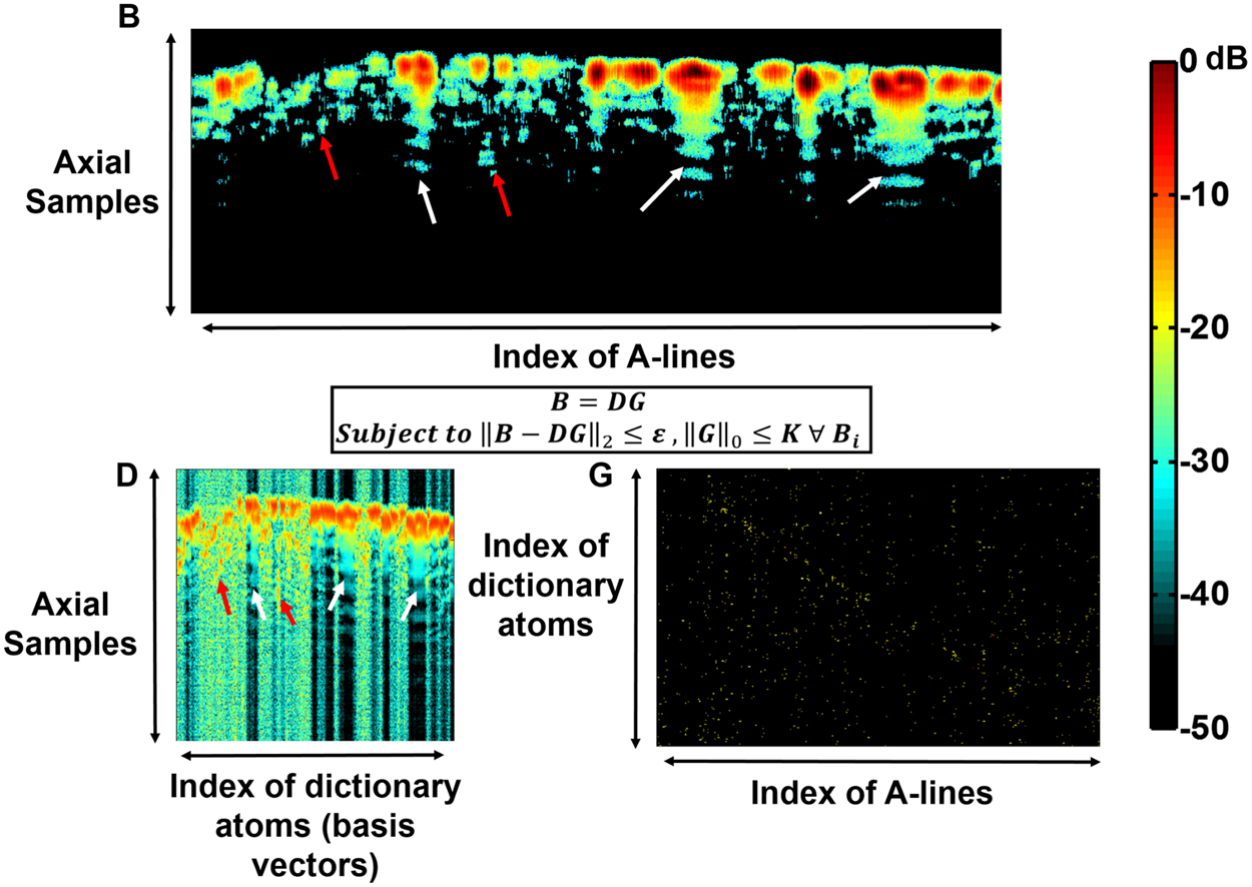
Decomposition of the raw B-scan (**B**) into Dictionary (**D**) and Coefficient (**G**) matrices. Note that **G** is sparse and contains very few significant coefficients.

Here, *K* is the number of dictionary atoms (basis vectors) that comprise each A-line. *D* and *G* represent the dictionary and sparse code matrices, analogous to the basis vectors and sparse weighting. ***ε*** represents the error of the coded signal with respect to the raw signal due to system noise. Using this approach, each A-line in *B* is decomposed or coded into a linear combination of basis vectors represented by the columns of *D* (Fig. 3) and each basis vector is known as an atom of the dictionary. As illustrated by the constraints in Eqn. (8), each A-line of *B* is a combination of at most *K* dictionary atoms. It is important to note that the atoms in *D* are linear combinations of the atoms of the overcomplete basis (Supplementary section S1). Although multiple dictionary learning algorithms^17–19^ exist, the dictionary is learned using the K-SVD algorithm^10,11^ in this paper due to its rapid convergence and simple implementation. The optimization process in the K-SVD algorithm is briefly explained below, and additional details can be found in the literature^10,11^. The algorithm is illustrated in Fig. 2c and in pseudo code as follows.
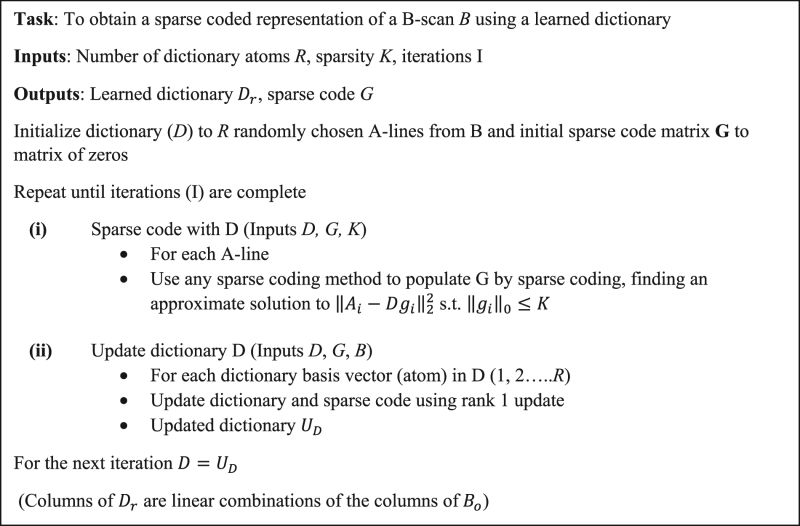

The algorithm proceeds using an alternating minimization procedure, where an initial dictionary *D* consisting of *R* atoms is assumed, and the data is sparsely coded using this dictionary. Each atom in the dictionary is subsequently updated to reduce the reconstruction error with respect to the input signals coded by that atom using a rank one approximation. This process is repeated iteratively until the error with respect to the input signal is lower than ***ε*** or until a specified number of iterations has elapsed. The parameters that have a bearing on the algorithms convergence are the number of atoms of the dictionary, the sparsity *K* and the error ***ε***. It is important to note that this is an unsupervised learning procedure, in that no information is provided *a-priori* to the algorithm about which signals actually contain reverberation.

Consequently, the dictionary atoms are linear combinations of the true signal and the reverberation, and these signals are further processed to remove the reverberant echoes while retaining the true signals. The dictionary atoms are normalized to have unit energy owing to the rank one approximation in the algorithm. Since the A-lines are sparse combinations of the dictionary basis vectors, it is sufficient to suppress the reverberation in each basis vector to obtain a reverberation-suppressed B-scan (Fig. 2b). An example of a learned dictionary and sparse code from a given B-scan is illustrated in Fig. 3. It may be observed that the learned dictionary (*D*) accurately captures the reverberant artifacts (white arrows) and signals of smaller amplitude (red arrows) and the matrix (*G***)** is sparse.

### Algorithm performance

A common initialization for the K-SVD algorithm, using randomly selected samples of the data itself as the initial dictionary, rather than an established basis such as the Fourier, cosine or wavelet bases, was used^10^. This initialization method has its origins in the K-SVD being a generalization of the K-means algorithm, which obtains feature/dictionary vectors using the expectation minimization algorithm—a specific instance of the more general alternating minimization method. The K-SVD algorithm has a natural noise reducing property (Figure S1), since it seeks to find features common to the coded signals during the update step.

Naturally uncorrelated white Gaussian noise is reduced first during the sparse coding process and then during the rank one update step described in preceding section. Applying the K-SVD algorithm with a well-tuned dictionary size and sparsity can further reduce the noise floor lower while retaining useful signal (Figure S1a). The performance of the algorithm was characterized by varying the different parameters such as dictionary size, sparsity *K*, and number of iterations for which the algorithm was executed. The algorithm performance in coding the signal was assessed by measuring the intensity of the error between the signals reconstructed from the learned dictionary against the raw data, normalized to the maximum signal amplitude. The error was averaged over 300 B-scans, each of which contains 3600 A-lines.

### Reverberation suppression

From the formulations discussed in the preceding sections (Eqns (2–7)), it is known that the reverberation and the true signal can both be expressed as sparse combinations of the shifted transducer impulse response. Additionally, it is known that the amplitudes of reverberant echoes decrease with each successive reflection (Eqns (2–4)). The number of the reverberant echoes (*N*) (Eqns (5–7)) is dependent on the acoustic impedance mismatch and varies spatially. However, we note from our model (Eqns. (6, 8, Supplementary section S1)) that choosing a subset of signals from each dictionary atom can suppress the reverberation. To extract this subset, we assume that the strongest signal in each basis vector in the dictionary is the ‘true’ signal originating from the transient thermoelastic expansion at the PAM focus.

**Figure 4:**
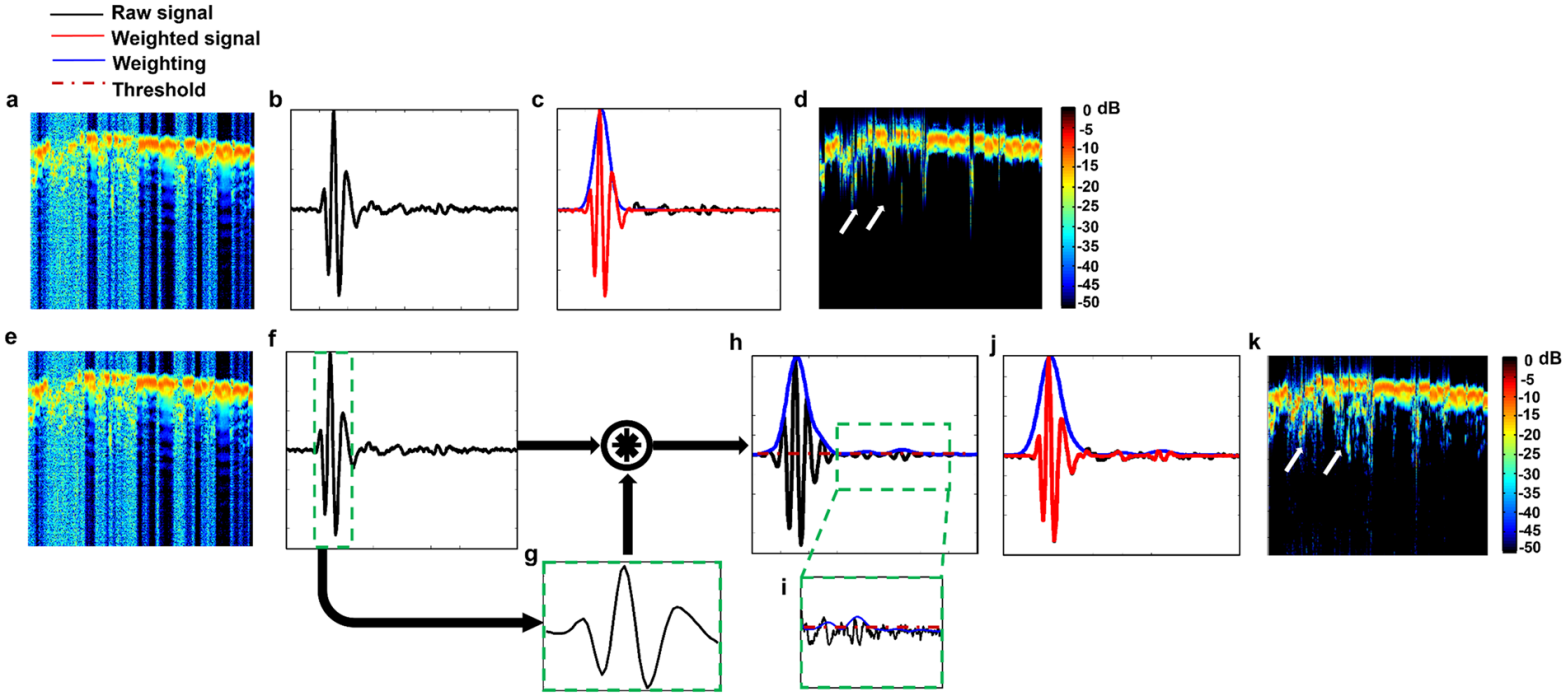
(**a**,**e**) Dictionary learned from B-scan (**b**,**f**) Representative atom (**c**) Maximum window weighted atom, (**d**) Maximum window weighted dictionary, (**g**) Signal of maximum intensity isolated from representative atom (**f**) (green box). (**h**) Cross correlation of (**f**) and (**g**). (**i**) Zoomed inset showing small signals retained under threshold (**j**) Correlation weighted atom (**k**) Correlation weighted dictionary. Purple line shows threshold, blue line shows weighting function, red line shows signal after weighting is applied and raw signal is shown in black.

However, isolating only the strongest signal may cause loss of signals from smaller vessels or regions of the tissue containing fewer optical absorbers. Hence, we cross-correlate each atom (Fig. 4f) with the strongest signal present in it (Fig. 4g) to find all similar signals (Fig. 4h) and obtain a weighting function to suppress reverberation. The envelope of the cross correlation is thresholded (Fig. 4h,i) and used to suppress the reverberation. As shown in Fig. 4k, the correlation weighting suppresses the reverberation in the resultant dictionary. It can be observed from Fig. 4c and j that the correlation window retains more of the signals possessing smaller amplitude. This is confirmed in the contrasting figures (Fig. 4d,k) where the signals of smaller amplitude are retained (white arrows). The reverberation suppression procedure is illustrated in Fig. 2d,e, and in pseudo-code as follows.
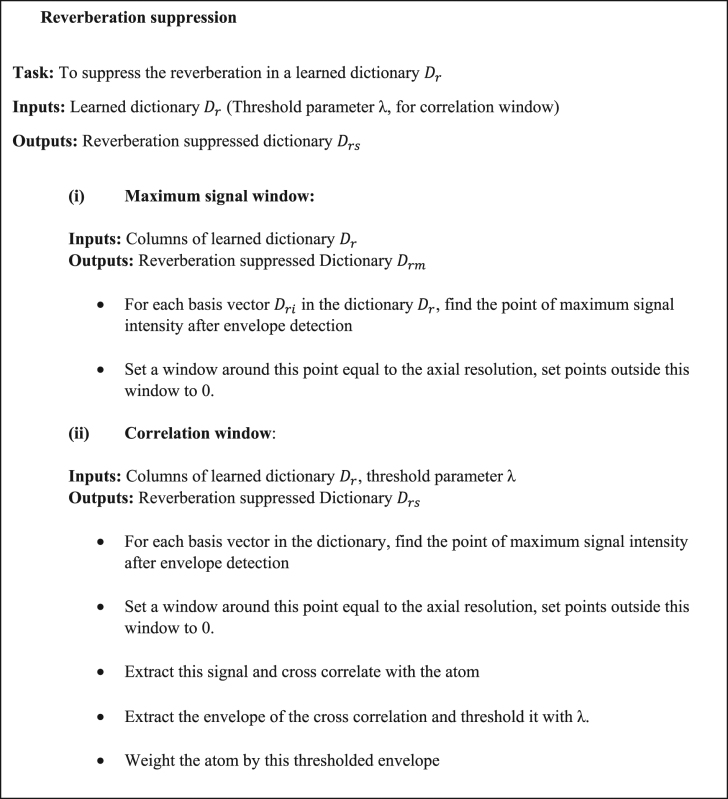

**Figure 5:**
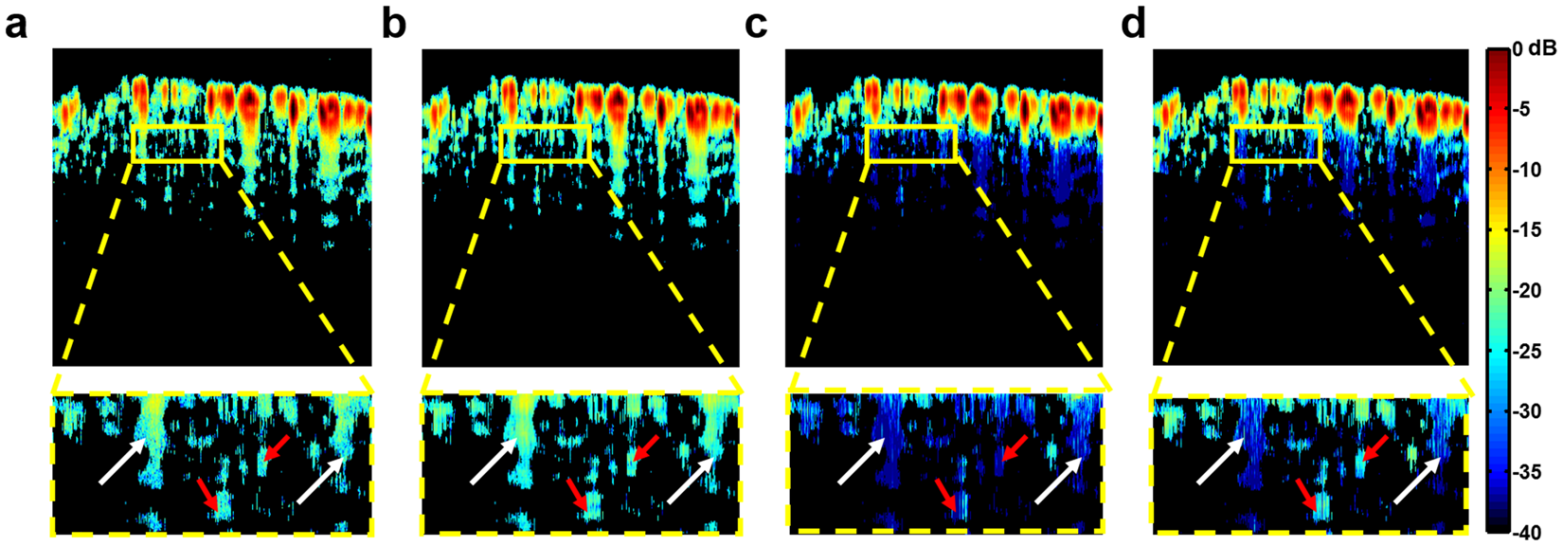
(**a**) Raw signal on masked area. (**b**) Dictionary coded signal overlaid on true signal area. (**c**) Maximum windowed signal overlaid on true signal area. (**d**) Correlation windowed signal overlaid on true signal area. White arrows denote reverberant echoes, and red arrows denote true signals of smaller amplitude. As shown in the zoomed insets, the signals of smaller amplitude are retained in the correlation-windowed signal (**d**), compared to (**c**).

This was confirmed by overlaying the signals constructed from the learned dictionary, maximum window suppressed signal, and correlation window suppressed signal on a binary mask constructed from the raw B-scan. The mask was constructed by choosing regions of the B-scan image which are above −40 dB relative to the maximum. The ground truth B-scan (Fig. 5a) overlaid on the same masked area is shown for reference. No significant difference can be observed between Fig. 5a and b, illustrating that the dictionary is correctly capturing pertinent signals. Additionally, it can be observed from Fig. 5c and its corresponding zoomed inset that although the reverberant artifacts (white arrows) are suppressed, there is also loss of signals of small amplitude (red arrows). However, when the correlation weighted window is used (Fig. 5d) the signals of small amplitude are retained, while the reverberation is suppressed.

### *In vitro* validation

**Figure 6:**
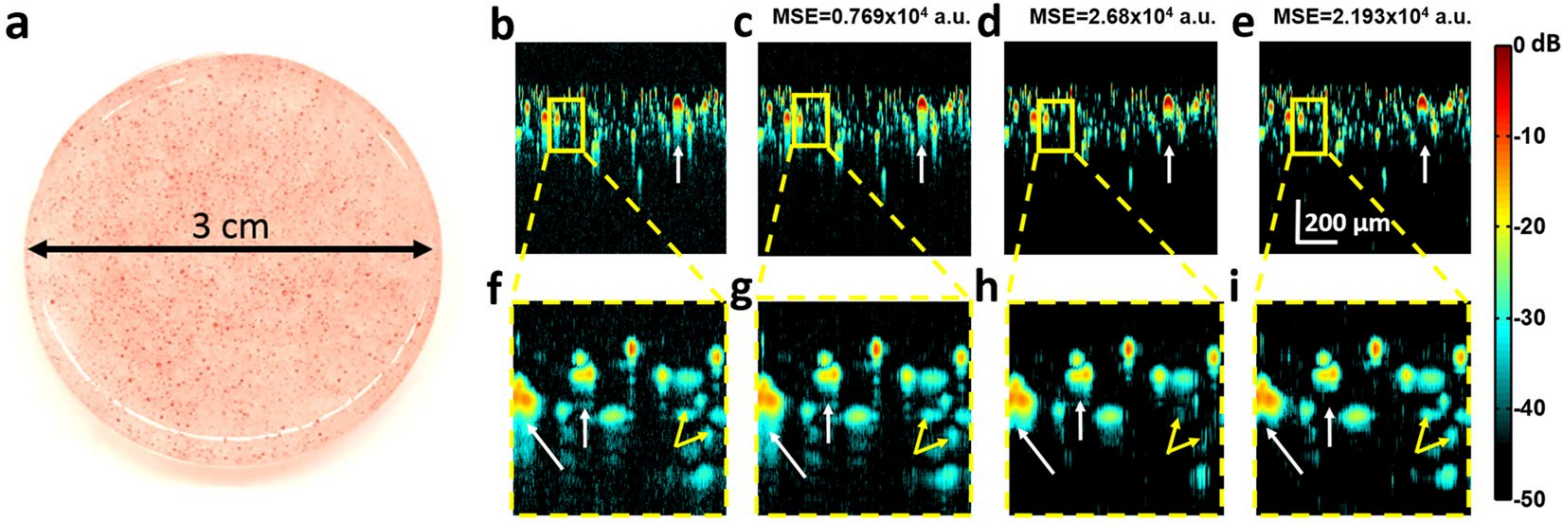
(**a**) *In vitro* PDMS phantom. (**b**) Representative B-scan (Raw signal) (**c**) Dictionary Coded B-scan, (**d**) Reverberation removed by maximum signal window. (**e**) Reverberation removed by correlation window. (**f**–**i**). Corresponding zoomed insets of (**b**–**e**). Removed reverberation is indicated by the white arrow in zoomed insets (**h**,**i**), retained small signals indicated by the yellow arrows.

For the *in vitro* experiments, data were acquired using 6-μm-diameter dyed polystyrene beads (Polysciences Inc.) embedded in transparent PDMS (Sylgard 184, Dow-Corning), as shown in Fig. 6a. Since PDMS is optically transparent, this allows us to have a known distribution of optical absorbers without potential signal from the background. The data were acquired using the setup shown in Fig. 2a. A representative B-scan of the results is shown in Fig. 6b. It may be observed in Fig. 6c that the signal and reverberation are coded accurately using the learned dictionary which is confirmed in the corresponding zoomed insets. The result of different windowing methods were compared in Fig. 6d,e. It may be observed that the reverberation signal is suppressed in both figures and their corresponding zoomed insets Fig. 6h,i (white arrows).

Additionally, it may be observed from Fig. 6h that some of the signals of lower amplitude (yellow arrows) are also lost compared to the coded signal when the maximum signal window is used. It may be observed in Fig. 6i that a larger number of signals of lower intensity (yellow arrows) are retained using the correlation windowing compared to the maximum signal window used in Fig. 6h. The difference in the retained signal is quantified using the mean square error (MSE). The MSE was calculated with respect to the raw signal (Fig. 6b) after coding with the learned dictionary (Fig. 6c), suppression with the maximum signal window (Fig. 6d), and the correlation weighted window (Fig. 6e). It may be observed, as expected, that there is a residual error after coding with the learned dictionary due to the noise suppression property of the algorithm (See Methods, *Tuning of algorithm parameters*). In addition, it may be observed that the error with respect to the raw data is smaller when the correlation window is used to suppress reverberation, rather than the maximum signal window, as more of the signals of smaller amplitude are retained. Furthermore, it may be observed from (Fig. 6h,i) that some of the noise retained after the coding is further suppressed. This is due to the consideration of only the signal of maximum amplitude and the threshold applied to the envelope of the correlation as discussed in the preceding section (See Fig. 5c,d).

### *In vivo* validation

**Figure 7:**
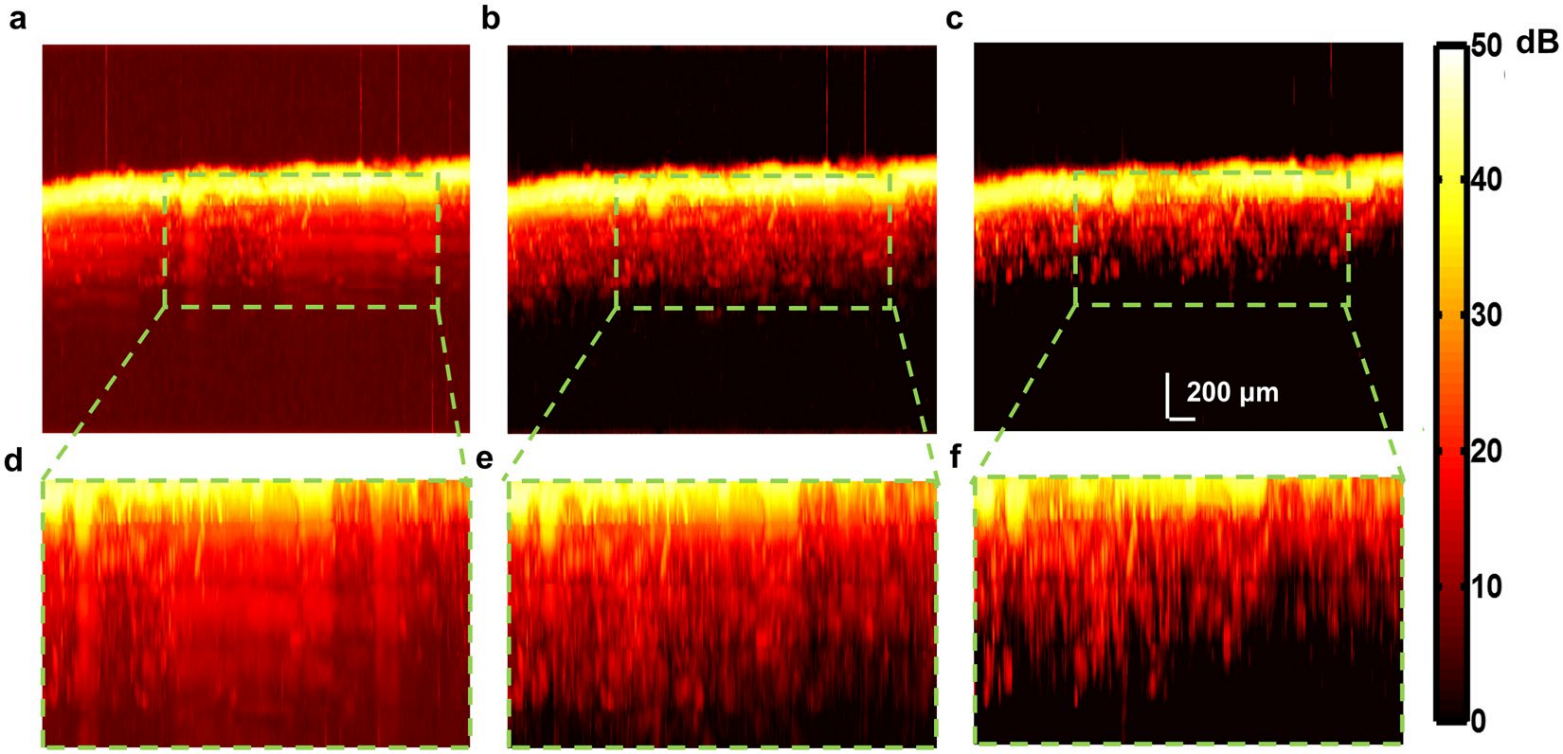
XZ projection PAM image of mouse cortical vasculature (**a**) Using raw signal (**b**) Applying correlation window directly to B-scans (**c**) Applying correlation window to dictionary learned from B-scans. Reverberation is suppressed but cortical vessels are more clearly delineated when the window is applied to the dictionary as seen in corresponding zoomed insets (**d**–**f**).

The algorithm was also validated *in vivo* in PAM images of the mouse brain (See Methods, *Animal preparation*), using the setup shown in Fig. 2 (See Methods, *Photoacoustic Microscopy*). The focal plane was gradually translated axially from 50 to 450 µm beneath the cortical surface with a 100-µm step size. A composite B-scan was constructed from the data ±50 μm about the focal plane of each of the five datasets to evaluate the suppression of reverberation. The maximum amplitude projection (MAP) image in the XZ plane was constructed from the raw composite B-scans and the composite B-scan obtained after processing with our algorithm. It may be observed from Fig. 7a and its corresponding zoomed inset Fig. 7d that the reverberant signal from superficial cortical layers completely obscures the vasculature at deeper layers. The performance of the algorithm was also compared with and without the use of dictionary learning. The XZ projection was also constructed after applying the windowing procedure directly to the B-scan with identical parameters (see Fig. 7b). It may be observed from the figure that the vessels do not appear as distinct as with the use of DL. Additionally, it may be observed that the contrast in the raw XZ-projection image (Fig. 7a) is poor due to the relatively high noise floor. The noise reducing capability of the K-SVD reduces this noise and consequently increases contrast as is seen in Fig. 7c and its corresponding zoomed inset (Fig. 7f). The zoomed insets Fig. 7(d–f) show the enhanced suppression of reverberation and clear delineation of microvessels penetrating the mouse cortex after the the use of DL. This was confirmed from the B-scans (Figure S4), where DL is observed to reduce noise, enhance the reverberation suppression, improve contrast and consequently improve the delineation of penetrating cortical microvessels. We quantify this improvement in performance using the mean intensity of the error with respect to the original signal with, and without dictionary learning (Figure S2) in a region of interest deep in tissue (Figure S2 (yellow box)) over 300 B-scans.

**Figure 8:**
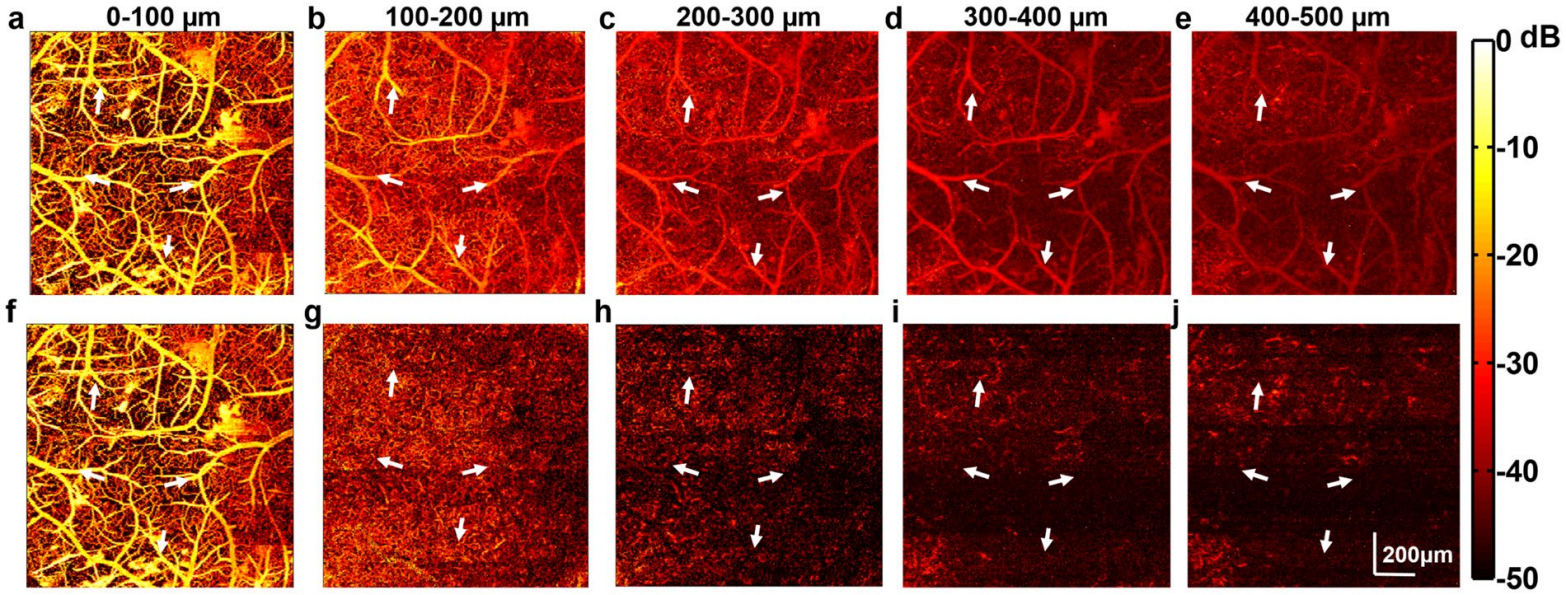
Representative MAP images before and after processing. (**a**–**e**) show the images before processing with reverberant artifacts denoted by arrows. (**f**–**j**) show the images after processing. The MAP images were generated using data ±50 µm about the focal plane (depth indicated in the figure). The arrows indicate the ghosting artifacts in the MAP images from reverberation.

Additionally, the suppression of reverberant artifacts was also verified in the XY-projection image from data ±50 μm about the focal plane at each depth. Reverberation from superficial vessels manifests as ‘ghosting’ creating false copies on images deeper in the mouse cortex as shown in Fig. 8(a–e), where the arrows indicate the position of spurious ghosting artifacts. After reverberation removal, the obscured microvasculature at deep layers becomes visible. However, the algorithm is still limited by physical processes such as optical scattering and absorption losses. The larger vessels and dense superficial vasculature absorb most of the energy from the optical excitation, which leads to shadowing under the major vessels at increased depths as is seen in Fig. 8h. Furthermore, optical scattering also reduces the energy of the excitation delivered to deeper tissue, which also reduces the available signal at increased depths as seen in Fig. 8i,j.

## Discussion

In this paper, an algorithm based on DL to remove reverberation in PAM was presented. Dictionary learning is a powerful algorithmic tool that has been used in many applications, including denoising^11,20^, blind source separation^21^, super-resolution^22^, deblurring^23^, and compressed sensing. Although K-SVD offers an elegant and useful solution, it is still difficult to tune properly on larger datasets due to the relatively long run time (20 seconds for each B-scan with an A-line interval of 0.83 µm and a sparsity of 5). Fortunately, online and minibatch methods^17^ of DL have been developed, which can reduce the run time by learning dictionaries while the data is acquired. The use of dictionary learning is invaluable as it allows for an abstraction of pertinent signal features which are common among A-lines. Using the windowing methods on the B-scans directly leads to sub-par results, since we also have to contend with noise. In fact, this case is subsumed in our model, since it leads to a trivial dictionary where every A-line is assumed as an atom and the sparse matrix is an identity matrix. However, a key advantage of the method is lost when processing the images this way, in that non-essential signals such as system noise are also introduced into the process, so while assigning the dictionary may be simple, the dictionary itself may be excessively noisy and contain information that is not relevant.

Additionally, if we have further information *a-priori* about regions of the vasculature that need to be included, or excluded, the dictionary could be learned using a supervised approach^18,24^. Implementation of these methods would allow coding and reverberation removal as the PAM data is being acquired rather than retrospectively on saved data as presented herein. Furthermore, extending the present study to multi-parametric PAM^16,25^ may enable depth-resolved quantification of blood perfusion, oxygenation and flow across different cortical layers. Combining these 3D-resolved functional parameters may ultimately enable label-free high-resolution imaging of cerebral oxygen metabolism in important disease models such as ischemic stroke, epilepsy and traumatic brain injury.

## Methods

### Tuning of Algorithm parameters

The performance of the algorithm was characterized with respect to variation in its parameters. Figure S2 shows the algorithm performance with varying sparsity and dictionary size. The sparsity represents the number of atoms (basis vectors) of the final learned dictionary that comprise each A-line of a B-scan. The lower the sparsity, the better the dictionary is able to reject noise and unwanted information. However, this can also increase the error of the coded signal with respect to the input signal, and increasing the number of dictionary atoms or algorithm iterations can compensate for error from low sparsity (Figure S2b). The computational complexity of the algorithm as a whole scales nonlinearly with both the number of atoms in the dictionary and the number of A-lines to be coded, but linearly with the number of iterations^26^ which is reflected in Figure S2c.

At first glance, it may appear that a larger number of iterations would reduce the error with respect to the raw data further and are hence more desirable. However, subsequent careful investigation (Figure S3) reveals that the reduction in error is primarily due to the dictionary adapting better to the noise in the signal, which indicates wasted computational effort due to increased computational time coding undesirable signal, and mitigates the noise suppressing advantage of the algorithm. Hence, we choose a sparsity of 5 with a dictionary size of 700 with a single iteration. The algorithm parameters were tuned and the same parameters were used for all subsequent experiments on *in vivo* and *in vitro* data. The noise suppression of K-SVD was characterized by assessing the noise floor from a 100 μm wide region of interest deep in the tissue (approx. 1 mm), where there is unlikely to be any useful signal from dictionary coded and raw PAM data. The noise floor was found to be 25.42 ± 0.05 dB below the maximum signal intensity (averaged over 300 B-scans) for the raw B-scans and 42.12 ± 0.15 dB below the maximum signal intensity.

### Photoacoustic microscopy

In our PAM system (Fig. 2a), a nanosecond-pulsed 559 nm laser (BX40-2-G, Edgewave) operating at the repetition rate of ~10 kHz is applied. Attenuated by a neutral density filter (NDC-50C-2M, Thorlabs), the beam is then filtered by an iris (SM1D12D, Thorlabs) and focused by a condenser lens (LA1608, Thorlabs). The focused beam is further filtered by a 50-μm-diameter pinhole (P50C, Thorlabs) prior to entering the microscope objective (M-10X, Newport), which is used to couple the beam into the single-mode optical fiber. To monitor the laser fluctuation, a beam sampler (BSF10-A, Thorlabs) is placed on the beam path to reflect a small portion (i.e., 5%) of the laser energy to a high-speed photodiode (FDS100, Thorlabs).

The other end of the single-mode fiber is mounted onto a 3-axis translational stage, which allows the adjustment of vertical position and 2D raster scan. Exiting from the single-mode fiber, the laser beam is collimated by an achromatic doublet (AC127-025- A, Thorlabs) and reshaped by an iris (SM05D5, Thorlabs), which is further focused using the identical achromatic doublet. A correction lens (LA1207-A, Thorlabs) is used to compensate for the optical aberration at the interface between the ambient air and water. The laser excitation light goes through the central hole of our customized ring-shaped ultrasonic transducer (center frequency: 35 MHz; 6-dB bandwidth: 70%), allowing the confocal alignment of the optical and acoustic foci to ensure the maximum sensitivity.

### Animal preparation

Male CD-1 mice (9–13 weeks old, Charles River Laboratories) were used for the studies. Under anesthesia, the hair in the mouse head was removed and a surgical incision was made in the scalp to expose the skull. The skull over the region of interest was removed using the established surgical procedure^27^. The mouse was anesthetized with 1.5% vaporized isoflurane, and its body temperature was kept at 37 °C using a heating pad (Omega, SRFG-303/10) and a temperature controller (Cole-Parmer, EW-89802-52). All experimental procedures were carried out in conformity with the laboratory animal protocol approved by the Animal Care and Use Committee at the University of Virginia.

## Electronic supplementary material

Supplementary Information

## References

[1] Wang LV, Hu S (2012). Photoacoustic tomography: *in vivo* imaging from organelles to organs. Science.

[2] Diebold GJ, Sun T (1991). Properties of potoacoustic waves in one, two, and three dimensions. Phys. Rev. Lett..

[3] Zhou Y, Yao J, L. VW (2016). Optical clearing-aided photoacoustic microscopy with enhanced resolution and imaging depth. Opt. Lett..

[4] Lediju Bell MA, Kuo NP, Song DY, Kang JU, Boctor EM (2014). *In vivo* visualization of prostate brachytherapy seeds with photoacoustic imaging. J. Biomed. Opt..

[5] Hojman E (2017). Photoacoustic imaging beyond the acoustic diffraction-limit with dynamic speckle illumination and sparse joint support recovery. Opt. Express.

[6] Burgholzer P, Sandbichler M, Krahmer F, Berer T, Haltmeier M (2016). Sparsifying transformations of photoacoustic signals enabling compressed sensing algorithms. Proc. SPIE.

[7] Haltmeier M, Berer T, Moon S, Burgholzer P (2016). Compressed sensing and sparsity in photoacoustic tomography. Opt. Express.

[8] Haq, I. U., Nagaoka, R., Siregar, S. & Saijo, Y. Sparse-representation-based denoising of photoacoustic images (2017).

[9] Candes, E. J., Romberg, J. & Tao, T. Robust uncertainty principles: exact signal reconstruction from highly incomplete frequency information. *IEEE Trans. Inf. Theory* **52** (2006).

[10] Aharon M, Elad M, Bruckstein A (2006). K-SVD: An Algorithm for Designing Overcomplete Dictionaries for Sparse Representation. Trans. Signal Process. IEEE.

[11] Mairal J, Elad M, Sapiro G (2008). Sparse representation for color image restoration. IEEE Trans. Image Process..

[12] Diebold GJ, Sun T, Khan MI (1991). Photoacoustic monopole radiation in one, two, and three dimensions. Phys. Rev. Lett..

[13] Donoho DL (2006). Compressed sensing. Ieee Trans. Inf. Theory.

[14] Tropp JA, Gilbert AC (2007). Signal recovery from random measurements via orthogonal matching pursuit. IEEE Trans. Inf. Theory.

[15] Chen SS, Donoho DL, Saunders MA (1998). Atomic Decomposition by Basis Pursuit. SIAM J. Sci. Comput..

[16] Cao R (2017). Functional and oxygen-metabolic photoacoustic microscopy of the awake mouse brain. Neuroimage.

[17] Mairal, J., Bach, F., Ponce, J. & Sapiro, G. Online dictionary learning for sparse coding. *Proc. 26th Int. Conf. Mach. Learn*. 1–8, 10.1145/1553374.1553463 (2009).

[18] Jiang Z, Lin Z, Davis LS (2013). Label consistent K-SVD: Learning a discriminative dictionary for recognition. IEEE Trans. Pattern Anal. Mach. Intell..

[19] Kreutz-delgado K (2003). Dictionary learning algorithms for sparse representation. Neural Comput..

[20] Mairal J, Bach F, Ponce J, Sapiro G, Zisserman A (2008). Discriminative learned dictionaries for local image analysis. IEEE Conf. Comput. Vis. Pattern Recognit..

[21] Turek, J. S., Elad, M. & Yavneh, I. Clutter Mitigation in Echocardiography Using Sparse Signal Separation. **2015** (2015). 10.1155/2015/958963 PMC4495184 26199622

[22] Yang J, Wright J, Huang TS, Ma Y (2010). Image super-resolution via sparse representation. IEEE Trans. Image Process..

[23] Das, R., Bajpai, A. & Venkatesan, S. M. Fast non-blind image deblurring with sparse priors. *Adv. Intell. Syst. Comput*. **459 AISC** (2017).

[24] Deng L, Yu D (2013). Deep Learning: Methods and Aopplications. Found. Trends Signal Process..

[25] Ning B (2015). Simultaneous photoacoustic microscopy of microvascular anatomy, oxygen saturation, and blood flow. Opt. Lett..

[26] Christensen, M. G. & Sturm, B. L. Comparison of Orthogonal Matching Pursuit Implementations. *20th Eur. Signal Process. Conf*. 220–224 (2012).

[27] Mostany, R. & Portera-Cailliau, C. A Craniotomy Surgery Procedure for Chronic Brain Imaging. *J. Vis. Exp*. 18–19, 10.3791/680 (2008). 10.3791/680 PMC2582844 19066562

