# Supplementary material for: Dictionary learning-based reverberation removal enables depth-resolved photoacoustic microscopy of cortical microvasculature in the mouse brain
Source: Sci Rep. 2018 Jan 17;8:985. doi: 10.1038/s41598-017-18860-3 (PMC5772684; doi:10.1038/s41598-017-18860-3)
Supplement: Supplementary file 1 — Supplementary Information [file 41598_2017_18860_MOESM1_ESM.pdf]

Supplementary Information.

Dictionary learning-based reverberation removal enables depth-resolved photoacoustic microscopy of cortical microvasculature in the mouse brain

Sushanth Govinahallisathyanarayana[1], Bo Ning[1,2], Rui Cao[1], Song Hu[1], John A. Hossack[1]

[1]Department of Biomedical Engineering, University of Virginia, Charlottesville, VA 22908

[2]Current address: Department of Biomedical Engineering, Johns Hopkins University, Baltimore, MD 21205

Corresponding authors: Song Hu (Photoacoustic microscopy) songhu@virginia.edu,

John A. Hossack (Ultrasound) jh7fj@.virginia.edu

Supplementary section 1.

Data in PAM are acquired as a two-dimensional ensemble of A-lines using a raster scan.

Each A-line can be modelled as follows,

$$A_i = H \otimes \left(I_{D_i \text{ x } D_i} | S_{D_i \text{ x } D_r} \right) m_i \quad \text{(S1)}$$

where $I_{D_i \text{ x } D_i}$ is an identity matrix of dimensionality equivalent to the imaging depth D_i, H is the transducer receive mode impulse response, and $\otimes$ denotes convolution along the axial dimension with each column of the matrix. Note that S is a matrix, the columns of which span the possible locations of reverberant signal, with each column being a sparse vector as described in **Eqn.** (**6**). m_i is a weighting vector which selects the locations of the true signal and reverberant signal in the A-line and determines their contribution to A_i, within the imaging depth D_i. D_r denotes the number of possible combinations of a pair of interfaces with normalized acoustic reflectances α_i, α_j. It may be observed that the number of such possible combinations exceeds the number of samples in the imaging depth, leading to an over-complete basis, and increasing the sparsity of the vector m_i. Thus, from (**Eqn.** (**S1**)) a B-scan (B) can be expressed as the product of an over complete basis B_o with a larger number of basis vectors than potential sample points and sparse matrix M comprised of the sparse vectors m_i. Where

$$B_o = H \otimes \left(I_{D_i x D_i} \mid S_{D_i x D_r} \right) \quad \text{(S2)}$$

$$B = B_o M \quad \text{(S3)}$$

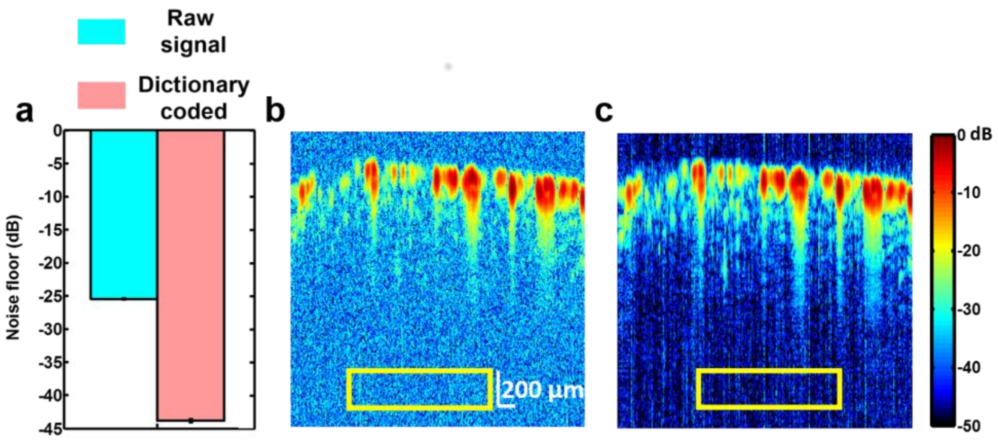

Supplementary figure S1 | Noise suppression built in to K-SVD. (a) Noise floor relative to maximum signal intensity, measured by average signal intensity in deep tissue (yellow box (b) and (c)) N=300 for each bar. Error bars represent standard deviation. (b) Representative raw B-scan (c) Dictionary coded B-scan (700 atoms 1 iteration)

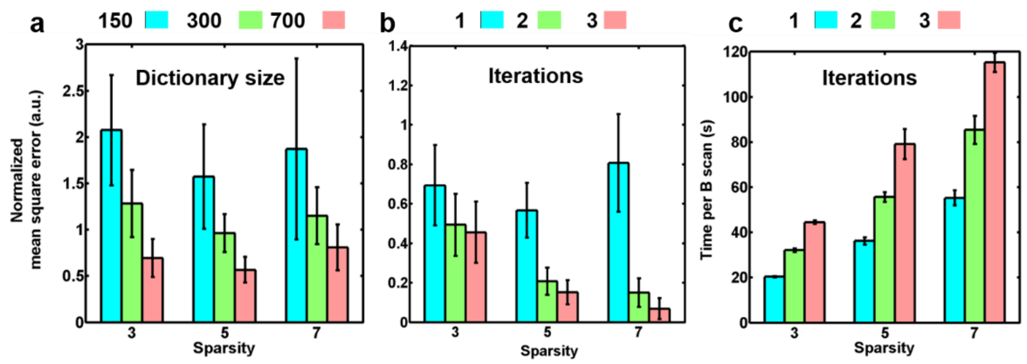

Supplementary figure S2 | (a) Representative variation in normalized mean square error between the raw signal and dictionary coded signal with increasing dictionary sizes and one iteration. (b) Representative reduction in normalized mean square error between the raw signal and dictionary coded signal for dictionary size of 700 atoms with increasing iterations. Error bars represent standard deviation, N=300 B scans for each bar. (c) Representative variation in execution time of algorithm per B scan with iterations and sparsity for dictionary size of 700 atoms N=10 B scans for each bar. Error bars represent standard deviation

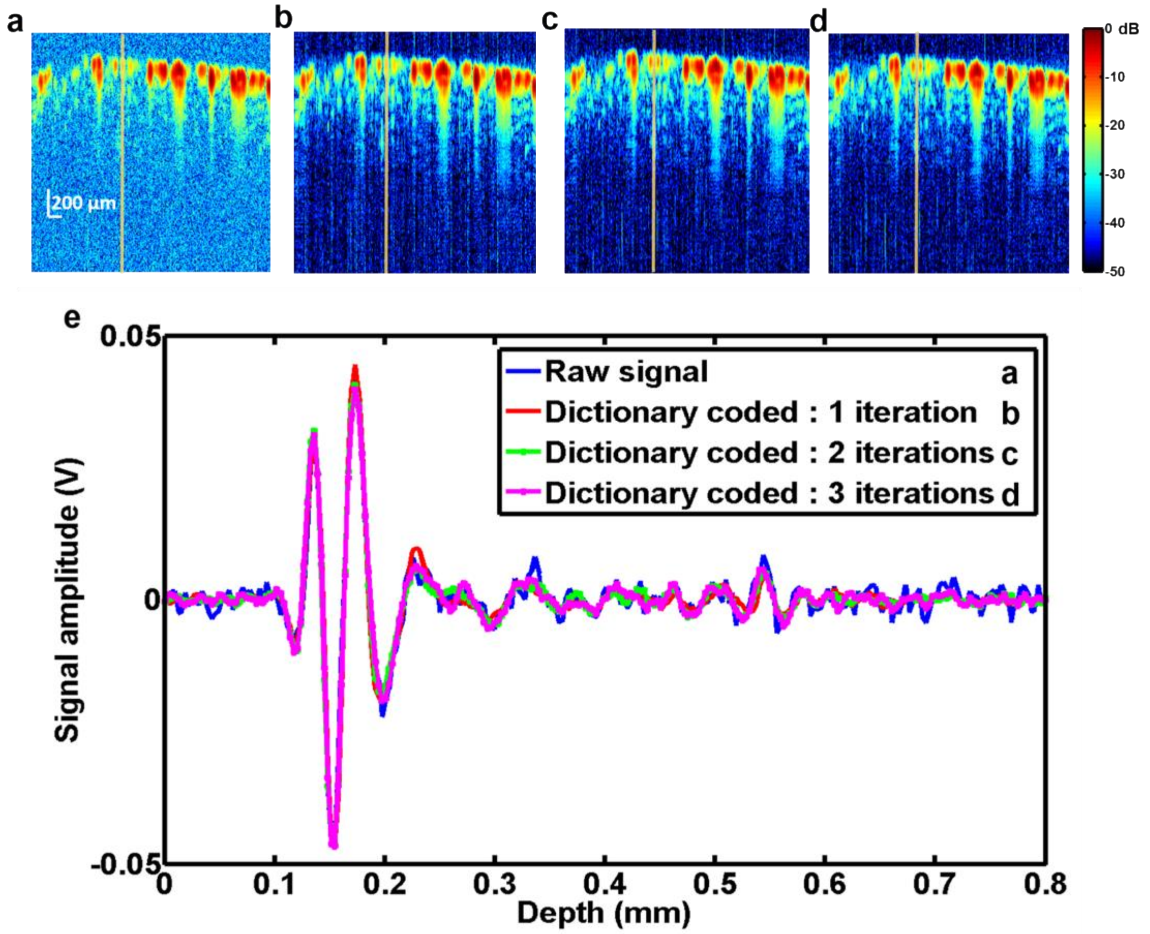

Supplementary figure S3 | (a) Representative variation in normalized mean square error between the raw signal and dictionary coded signal with increasing dictionary sizes and one iteration. (b) Representative reduction in normalized mean square error between the raw signal and dictionary coded signal for dictionary size of 700 atoms with increasing iterations. Error bars represent standard deviation, N=300 B-scans for each bar. (c) Representative variation in execution time of algorithm per B-scan with iterations and sparsity for dictionary size of 700 atoms N=10 B-scans for each bar. Error bars represent standard deviation.

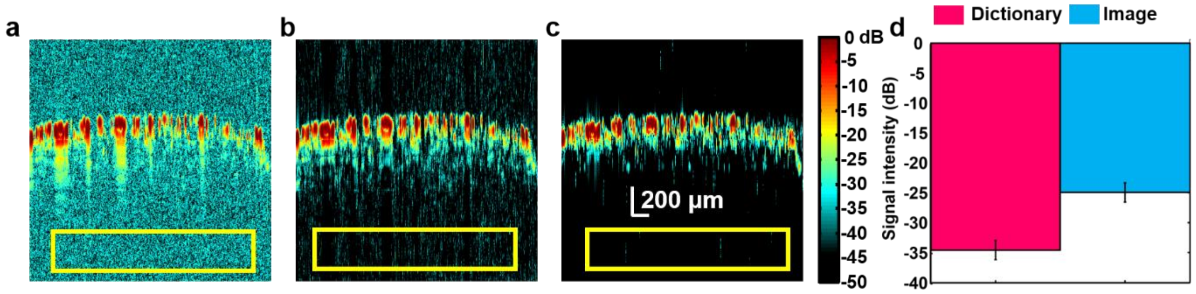

Supplementary Figure S4 | (a) Raw B-scan (b) Correlation window applied to B-scan (c) Correlation window applied to dictionary learned from B-scan. Region of interest (ROI) to quantify suppression (yellow box) (d) Difference in signal suppression in ROI (N=300 for each bar) error bars represent standard deviation.
